# C-reactive protein-to-albumin ratio as a biomarker in patients with sepsis: a novel LASSO-COX based prognostic nomogram

**DOI:** 10.1038/s41598-023-42601-4

**Published:** 2023-09-15

**Authors:** Xin Zhou, Shouzhi Fu, Yisi Wu, Zhenhui Guo, Wankang Dian, Huibin Sun, Youxia Liao

**Affiliations:** 1 Department of Emergency/Intensive Care Unit, Wuhan Third Hospital, Tongren Hospital of Wuhan University, No. 216 Guanshan Avenue, Hongshan District, Wuhan, Hubei China; 2 Cardiac Function Department, Asia Heart Hospital, Wuhan, China; 3 Department of 120 Emergency Center, Wuhan Third Hospital, Tongren Hospital of Wuhan University, Wuhan, China

**Keywords:** Immunological disorders, Computational biology and bioinformatics, Predictive medicine, Biomarkers

## Abstract

To develop a C-reactive protein-to-albumin ratio (CAR)-based nomogram for predicting the risk of in-hospital death in sepsis patients. Sepsis patients were selected from the MIMIC-IV database. Independent predictors were determined by multiple Cox analysis and then integrated to predict survival. The performance of the model was evaluated using the concordance index (C-index), receiver operating characteristic curve (ROC) analysis, and calibration curve. The risk stratifications analysis and subgroup analysis of the model in overall survival (OS) were assessed by Kaplan–Meier (K–M) curves. A total of 6414 sepsis patients were included. C-index of the CAR-based model was 0.917 [standard error (SE): 0.112] for the training set and 0.935 (SE: 0.010) for the validation set. The ROC curve analysis showed that the area under the curve (AUC) of the nomogram was 0.881 in the training set and 0.801 in the validation set. And the calibration curve showed that the nomogram performs well in both the training and validation sets. K–M curves indicated that patients with high CAR had significantly higher in-hospital mortality than those with low CAR. The CAR-based model has considerably high accuracy for predicting the OS of sepsis patients.

## Introduction

Sepsis is a potentially life-threatening complication of an infection with high short-term mortality^1,2^. In the past 10 years, the annual incidence of sepsis was 437/100,000, and the mortality rate was 17% in adults in developed countries. The annual incidence of severe sepsis is 270/100,000, and the mortality rate is 26%^3^. The incidence and mortality of sepsis are higher in developing and less developed countries^4^. In addition, treatment challenges and high costs pose a heavy burden on both society and the economy. Therefore, it is crucial to identify and predict the risk of death early in sepsis patients in order to evaluate the severity of the disease and make appropriate treatment decisions.

At present, diagnosis using the combination of multiple biochemical markers is a much pursued topic in sepsis research^5–7^. It is known that the development of sepsis is related to the imbalance of proinflammatory and anti-inflammatory responses^8,9^. In the early stage of sepsis, pro-inflammatory factors are released in large quantities by activated immune cells, which leads to a hyperimmune response and the generation of a cytokine storm^10^. As the disease progresses to the advanced stage, immune suppression or immune paralysis gradually occurs as anti-inflammatory cytokine levels increase in the presence of macrophage inactivation^11^. Clinically, serum C-reactive protein (CRP) is one of the most sensitive indicators for tissue damage and infection^12^. In addition, the relationship between inflammation and malnutrition is complex^13^. Inflammation during sepsis can lead to malnutrition, as it decreases appetite and alters metabolism^14,15^. On the other hand, malnutrition can predispose patients to infections, leading to exaggerated inflammation^16^. In general, malnourished individuals are at greater risk for developing serious infections, further exacerbating inflammation^17,18^. ALB (Serum albumin) has been widely used as an indicator of malnutrition in the clinical setting^19^. Recent studies have reported that CAR (C-reactive protein-to-albumin ratio) can serve as a new predictor for pneumonia, stroke, and cancers^20–23^.

Nevertheless, the role of CAR in predicting in-hospital mortality in sepsis patients is currently unclear. Therefore, this study is aimed to elucidate the association between CAR and 30-d (30-day) mortality in sepsis patients in the ICU and to further develop a prediction model for predicting the risk of in-hospital death in sepsis patients.

## Materials and methods

### Data collection

All data, including demographic characteristics, laboratory indicators, drugs, and complications, were extracted from the MIMIC-IV database. Patients confirmed with sepsis were eligible for enrollment. Sepsis was diagnosed according to the Third International Consensus Definitions for Sepsis (Sepsis-3). Briefly, patients with a suspected infection plus a Sequential Organ Failure Assessment (SOFA) score of ≥ 2 were diagnosed with sepsis. According to the criteria, a final total of 6414 eligible sepsis patients were included in this retrospective cohort study. The first author (XZ) obtained access to the database and was responsible for data extraction (certification number 53012064).

### Data extraction

In this study, CAR is the primary study variable. The following potential confounders were extracted: Demographics (Age, Gender, Race, Insurance, Marital status), drug treatment (Omeprazole, Cefazolin, Amoxicillin Clavulanic Acid, Ceftazidime, Pantoprazole, Miconazole, Linezolid, Metronidazole Flagyl, Heparin, Ciprofloxacin HCl, Erythromycin, Daptomycin, Dobutamine, Dopamine, Dexamethasone, etc.), comorbidities (Acute liver failure, Acute Kidney Injury, Acute Respiratory Failure, Heart Failure, Septic Shock, Venous Thrombosis, Stress Ulcer, Toxic Encephalopathy, Disseminated Intravascular Coagulation), laboratory indicators (Red Blood Cells, White Blood Cells, Platelets, Hemoglobin, Neutrophil, Lymphocyte, Lymphocyte percentage, Monocyte, Reticulocyte, Erythrocyte Specific Volume, Alanine Aminotransferase, Aspartate Transaminase, Creatine Kinase, Creatine Kinase-MB, Alkaline Phosphatase, C-reactive Protein, Serum Albumin, Globulin, Total Protein, Total Bilirubin, Direct Bilirubin, Indirect Bilirubin, Serum Glucose, Serum Creatinine, Blood Urea Nitrogen, Thrombin, Serum Potassium, Serum Sodium, Serum Chloride, Homocysteine, D-dimer, NT-proBNP (N-Terminal Pro-Brain Natriuretic Peptide), Troponin T, Lactate, Ferritin, Total Cholesterol, Triglyceride, High-density Lipoprotein, Low-density lipoprotein), primary outcome (Admission time, Discharge time, Death time), Simplified Acute Physiology Score II (SAPSII) and Sequential Organ Failure (SOFA). The endpoint event of this study was in-hospital death. All laboratory data were measured on the day of admission. If a variable was recorded more than once in the first 24 h, the value associated with the most severe disease was used. All codes used for comorbidities were based on the recorded ICD-9 (International Classification of diseases) codes and ICD-10 codes. The data mentioned above were extracted and cleaned by pg-Admin4 and R-studio. To avoid bias, continuous variables with over 50% missing data and categorical variables with less than 1% positive events were excluded, and missing values were imputed using multiple interpolations. Finally, the main study variable CAR was generated.

### Construction of prediction nomogram

The 6414 patients were randomly divided into the training set and validation set in a 7:3 ratio. The training set was used to determine the survival-related factors and to establish the nomogram. The validation set was used to verify the nomogram. A total of 43 variables related to the survival of sepsis patients were identified by univariate Cox regression. Least Absolute Shrinkage and Selection Operator (LASSO) regression was subsequently performed to further narrow down the above variables to 30. Variables with statistical significance were further included in the multivariate Cox regression analysis to determine independent predictors. An alignment diagram was established based on the independent risk factors affecting the prognosis of sepsis patients. The reliability of the model was verified by C-index, ROC curve, and calibration curve. Finally, patients were divided into high risk and low risk groups based on the constructed nomogram. Survival curves were generated using the K-M estimator, and the prognostic difference was determined by the log rank test. The optimal cut-off value of CAR was determined by median value, and this was used to divide the CAR into high risk and low risk groups. A subgroup analysis was performed to investigate the effect of CAR on different subgroups.

### Statistical analysis

Continuous variables with normal distribution are presented as mean ± standard deviation (Mean ± SD), and those without normal distribution are expressed as median and interquartile range [M (Q1, Q3)]. Variables with normal distribution and homogeneity of variance were compared between the two groups using the Student’s *t*-test; otherwise, they were compared using the Mann–Whitney U-test. Additionally, categorical data are expressed as total number and composition ratio [n (%)] and the difference between groups was compared using Pearson χ^2^ test and Fisher’s exact test. All tests were two-tailed and a *P* < 0.05 was considered statistically significant. All statistical analyses were performed using R 4.2.1, and SPSS 22.0.

## Results

### General patient data

**Figure 1:**
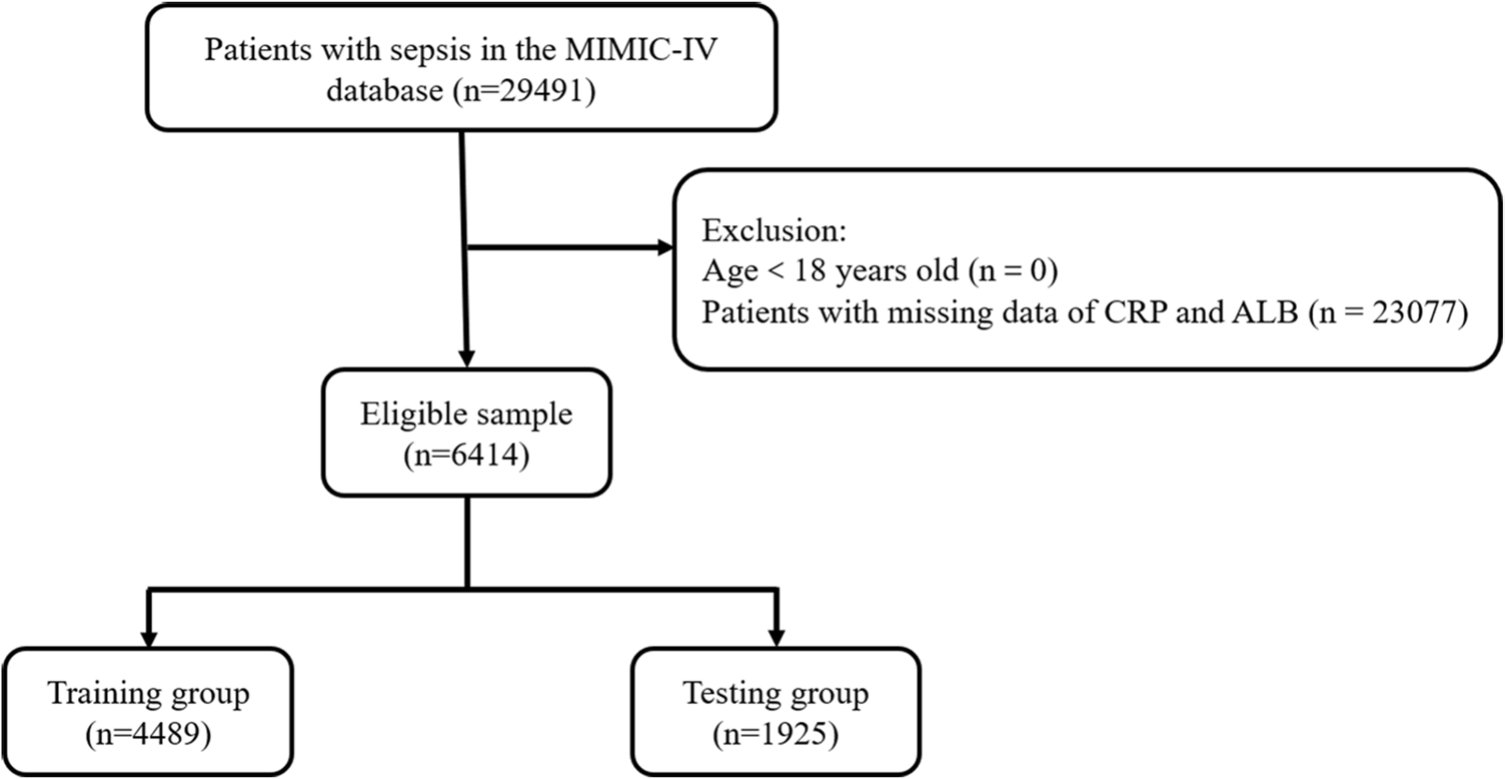
The flow diagram of study sample selection. *CRP* C-reactive protein, *ALB* serum albumin.

The patient selection flow chart is shown in Fig. 1. Of the 6414 sepsis patients (3500 males and 2914 females) included in this study, 4489 were assigned to the training set and 1925 were assigned to the validation set. The vast majority of patients were Caucasian and married or single, and only a minority had Medicaid insurance. During hospitalization, 61.6% of the patients developed acute renal failure, and 44.81% of the patients developed acute respiratory failure. In the training set, the mean patient age was 62.53 years and the average length of hospital stay was 9.8 days. The mean values of CAR were 17.5 and 17.56 in the training and validation sets, respectively. The in-hospital mortality rate was 3.24% (208/6414), 3.01% (135/4489), 3.79% (73/1925) in the total, training, and validation cohorts, respectively (log-rank test, training vs. validation cohort: p = 0.104). There were no significant differences in most clinical characteristics between the training and the validation cohorts. The baseline characteristics of all patients are summarized in Table 1 and Supplement Table 1.

**Table 1:** Basic characters.

Factors	Define	Train (N = 4489)	Test (N = 1925)	All (N = 6414)	t/Z (p)
Age		62.53 ± 15.66	61.77 ± 15.67		1.776 (0.076)
Marriage	Married	2124 (47.32)	903 (46.91)	3027 (47.19)	0.417 (0.812)
	Single	1510 (33.64)	642 (33.35)	2152 (33.55)	
	other	855 (19.05)	380 (19.74)	1235 (19.25)	
Gender	Male	2457 (54.73)	1043 (54.18)	3500 (54.57)	0.166 (0.684)
	Female	2032 (45.27)	882 (45.82)	2914 (45.43)	
Race	White	2930 (65.27)	1237 (64.26)	4167 (64.97)	0.605 (0.437)
	Other	1559 (34.73)	688 (35.74)	2247 (35.03)	
Insurance	Medicaid	418 (9.31)	169 (8.78)	587 (9.15)	0.573 (0751)
	Medicare	1844 (41.08)	787 (40.88)	2631 (41.02)	
	Other	2227 (49.61)	969 (50.34)	3196 (49.83)	
ALP (IU/L)		108.15 ± 109.98	103.55 ± 84.46		1.817 (0.069)
ALT (IU/L)		55.27 ± 225.07	59.4 ± 326.3		−0.585 (0.559)
AST (IU/L)		78.42 ± 474.07	69.16 ± 313.73		0.786 (0.432)
Glucose (mmol/l)		137.12 ± 81.08	136.37 ± 78.84		0.342 (0.732)
BUN (mg/dL)		24.53 ± 18.19	23.57 ± 17.46		1.969 (0.049)
Chloride (mEq/L)		101.67 ± 5.18	101.39 ± 5.43		1.941 (0.052)
Creatine kinase (IU/L)		491.52 ± 3919.28	491.97 ± 4443.74		−0.004 (0.997)
Creatine kinase-MB (IU/L)		8.51 ± 29.81	8.58 ± 27.2		−0.093 (0.926)
Serum creatinine (mg/dL)		1.41 ± 1.55	1.34 ± 1.34		1.664 (0.096)
Hemoglobin (g/l)		12.31 ± 2.21	12.3 ± 2.2		0.239 (0.811)
Hematocrit (%)		37.01 ± 6.24	37.05 ± 6.22		−0.226 (0.822)
HDL (mmol/L)		48.56 ± 20.52	49.29 ± 21.22		−1.301 (0.193)
Lymphocyte (%)		19.03 ± 12.09	19.23 ± 11.95		−0.612 (0.541)
Monocyte (%)		5.98 ± 3.3	6 ± 3.14		−0.259 (0.796)
Neutrophil (%)		71.23 ± 14.4	71.16 ± 14.18		0.173 (0.863)
NT-proBNP (pg/mL)		5386.83 ± 9616.44	5304.75 ± 9607.51		0.313 (0.754)
Platelet (K/μL)		245.01 ± 113.35	250.44 ± 116.73		−1.745 (0.081)
Potassium (mEq/L)		4.27 ± 0.69	4.26 ± 0.72		0.523 (0.601)
Sodium (g/L)		138.39 ± 4.42	138.26 ± 4.74		1.07 (0.285)
Total bilirubin (mg/dL)		1.05 ± 2.64	0.96 ± 2.28		1.258 (0.208)
Total cholesterol (mEq/L)		167.32 ± 57.75	168.06 ± 64.35		−0.452 (0.651)
Total protein (g/L)		6.56 ± 1.06	6.57 ± 1.05		−0.166 (0.868)
Triglyceride (mg/day)		158.82 ± 209.34	166.94 ± 229.56		−1.382 (0.167)
WBC (K/μL)		9.81 ± 7.72	10.12 ± 13.86		−1.157 (0.247)
Lactate (mmol/l)		2.02 ± 1.56	2.02 ± 1.64		−0.061 (0.952)
Ferritin (ng/mL)		633 ± 2683.67	649.01 ± 4185.73		−0.183 (0.855)
RBC (m/μL)		4.1 ± 0.74	4.12 ± 0.73		−0.67 (0.503)
SAPSII		36.7 ± 13.44	36.59 ± 13.77		0.308 (0.758)
SOFA		5.87 ± 3.69	5.88 ± 3.79		−0.087 (0.931)
CRP (mg/L)		56.42 ± 70.49	56.25 ± 71.90		0.900 (0.928)
ALB (g/L)		3.66 ± 0.74	3.66 ± 0.75		−0.244 (0.807)
CAR		17.5 ± 23.97	17.56 ± 24.51		−0.09 (0.929)
Time		9.8 ± 12.8	9.51 ± 12.72		0.827 (0.409)
Acute kidney failure	No	1723 (38.38)	740 (38.44)	2463 (38.4)	0.002 (0.965)
	Yes	2766 (61.62)	1185 (61.56)	3951 (61.6)	
Toxic encephalopathy	No	3538 (78.81)	1511 (78.49)	5049 (78.72)	0.083 (0.773)
	Yes	951 (21.19)	414 (21.51)	1365 (21.28)	
Acute liver failure	No	4346 (96.81)	1866 (96.94)	6212 (96.85)	0.064 (0.800)
	Yes	143 (3.19)	59 (3.06)	202 (3.15)	
Acute respiratory failure	No	2513 (55.98)	1027 (53.35)	3540 (55.19)	3.770 (0.052)
	Yes	1976 (44.02)	898 (46.65)	2874 (44.81)	
Heart failure	No	4426 (98.6)	1903 (98.86)	6329 (98.67)	0.700 (0.403)
	Yes	63 (1.4)	22 (1.14)	85 (1.33)	
Stress ulcer	No	4402 (98.06)	1878 (97.56)	6280 (97.91)	1.670 (0.196)
	Yes	87 (1.94)	47 (2.44)	134 (2.09)	
Septic shock	No	3282 (73.11)	1395 (72.47)	4677 (72.92)	0.283 (0.594)
	Yes	1207 (26.89)	530 (27.53)	1737 (27.08)	
Cefazolin	No	3143 (70.02)	1379 (71.64)	4522 (70.5)	1.702 (0.192)
	Yes	1346 (29.98)	546 (28.36)	1892 (29.5)	
Ceftazidime	No	3746 (83.45)	1615 (83.9)	5361 (83.58)	0.197 (0.657)
	Yes	743 (16.55)	310 (16.1)	1053 (16.42)	
Ceftriaxone	No	3138 (69.9)	1318 (68.47)	4456 (69.47)	1.311 (0.252)
	Yes	1351 (30.1)	607 (31.53)	1958 (30.53)	
Pantoprazole	No	1565 (34.86)	686 (35.64)	2251 (35.1)	0.354 (0.552)
	Yes	2924 (65.14)	1239 (64.36)	4163 (64.9)	
Miconazole	No	3401 (75.76)	1497 (77.77)	4898 (76.36)	2.995 (0.083)
	Yes	1088 (24.24)	428 (22.23)	1516 (23.64)	
Linezolid	No	3954 (88.08)	1721 (89.4)	5675 (88.48)	2.305 (0.129)
	Yes	535 (11.92)	204 (10.6)	739 (11.52)	
Metronidazole Flagyl	No	2939 (65.47)	1323 (68.73)	4262 (66.45)	6.407 (0.011)
	Yes	1550 (34.53)	602 (31.27)	2152 (33.55)	
Ciprofloxacin HCl	No	2322 (51.73)	1013 (52.62)	3335 (52)	0.434 (0.51)
	Yes	2167 (48.27)	912 (47.38)	3079 (48)	
Erythromycin	No	4121 (91.8)	1771 (92)	5892 (91.86)	0.071 (0.791)
	Yes	368 (8.2)	154 (8)	522 (8.14)	
Heparin	No	107 (2.38)	34 (1.77)	141 (2.2)	2.388 (0.122)
	Yes	4382 (97.62)	1891 (98.23)	6273 (97.8)	
Dobutamine	No	4286 (95.48)	1860 (96.62)	6146 (95.82)	4.416 (0.036)
	Yes	203 (4.52)	65 (3.38)	268 (4.18)	
Dopamine	No	4214 (93.87)	1801 (93.56)	6015 (93.78)	0.230 (0.632)
	Yes	275 (6.13)	124 (6.44)	399 (6.22)	
Dexamethasone	No	3856 (85.9)	1635 (84.94)	5491 (85.61)	1.016 (0.314)
	Yes	633 (14.1)	290 (15.06)	923 (14.39)	
Daptomycin	No	3978 (88.62)	1729 (89.82)	5707 (88.98)	1.983 (0.159)
	Yes	511 (11.38)	196 (10.18)	707 (11.02)	
Omeprazole	No	2363 (52.64)	1007 (52.31)	3370 (52.54)	0.058 (0.809)
	Yes	2126 (47.36)	918 (47.69)	3044 (47.46)	
Amoxicillin Clavulanic acid	No	3907 (87.03)	1667 (86.6)	5574 (86.9)	0.227 (0.634)
	Yes	582 (12.97)	258 (13.4)	840 (13.1)	
Status	No	4354 (96.99)	1852 (96.21)	6206 (96.76)	2.645 (0.104)
	Yes	135 (3.01)	73 (3.79)	208 (3.24)	

*ALP* alkaline phosphatase, *ALT* alanine aminotransferase, *AST* aspartate transaminase, *BUN* blood urea nitrogen, *HDL* high-density lipoprotein, *NT-proBNP* N-Terminal Pro-Brain Natriuretic Peptide, *RBC* red blood cell, *WBC* white blood cell, *SOFA* Sequential Organ Failure Assessment, *SAPS II* Simplified Acute Physiology Score II, *CRP* C-reactive protein, *ALB* serum albumin, *CAR* C-reactive protein/albumin ratio.

### CAR is an independent prognostic predictor

**Figure 2:**
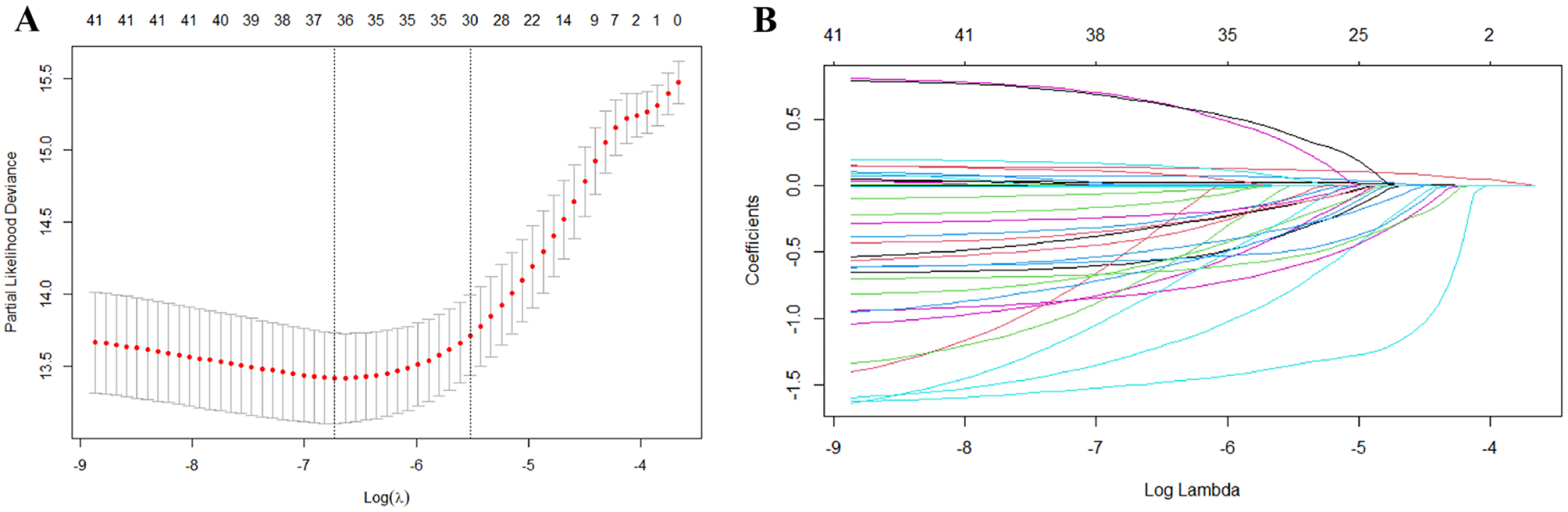
The LASSO logistic regression-based model for screening predictors: screening retention variables with the optimal value of lambda. (**A**) Tuning parameter (λ = 0.004832) selection using LASSO-type penalized logistic regression with tenfold cross-validation using minimum criteria. The partial likelihood deviance (binomial deviance) curve was plotted versus log (λ). Dotted vertical lines were drawn at the optimal values by using the minimum criteria and the one SE of the minimum criteria (the 1-SE criteria). (**B**) LASSO coefficient profiles of the thirty variables of the radiomic features. A coefficient profile plot was plotted versus the log (λ). Each colored line represents the coefficient of each feature. *LASSO* least absolute shrinkage and selection operator.

A total of 88 covariates with significant differences (*P* < 0.05) were identified by univariate COX regression analysis (Table 2). The results indicated that CAR was significantly associated with in-hospital mortality (HR, 1.01; 95% CI, 1.01–1.01; *P* < 0.001). In addition, 30 predictors were identified by LASSO regression analysis (Fig. 2). These predictors included age, race, insurance, marital status, neutrophil, lymphocyte, lactate, ferritin, CAR, CK-MB, HDL, NT-proBNP, Cefazolin, Ceftazidime, Ceftriaxone, Ciprofloxacin HCl, Daptomycin, Dexamethasone, Metronidazole Flagyl, Amoxicillin Clavulanic Acid, Linezolid, Erythromycin, Miconazole, Dobutamine, Dopamine, Heparin, Pantoprazole, Omeprazole, SAPS II and SOFA. These variables were included in the multivariate Cox regression analysis, and the results showed that CAR was positively correlated with the risk of in-hospital death among sepsis patients. Notably, CAR might be an independent predictor of in-hospital death in sepsis patients (HR, 1.01; 95% CI, 1.00–1.01; *P* = 0.0071).

**Table 2:** Univariate and multivariable COX.

Factors	Univariate COX		Multivariable COX	
	HR (95%CI)	Z (P)	HR (95%CI)	Z (P)
Age	1.04 (1.03–1.05)	0	1.03 (1.02–1.04)	0
Gender	0.88 (0.63–1.23)	0.4569		
Marital status	1.48 (1.18–1.87)	0.0008	1.24 (0.95–1.6)	0.1119
Insurance	0.77 (0.61–0.97)	0.0287	0.71 (0.54–0.94)	0.0152
Race	0.61 (0.43–0.85)	0.0036	0.71 (0.5–1)	0.052
WBC (K/μL)	1 (0.98–1.01)	0.8169		
RBC (m/μL)	0.78 (0.64–0.95)	0.0138		
Neutrophil (%)	1.02 (1.01–1.04)	0.0014		
Monocyte (%)	0.96 (0.92–1.01)	0.153		
Lymphocyte (%)	0.96 (0.95–0.98)	0.0002		
AST (IU/L)	1 (1–1)	0.7419		
ALP (IU/L)	1 (1–1)	0.1932		
ALT (IU/L)	1 (1–1)	0.9982		
Creatine kinase (IU/L)	1 (1–1)	0.9493		
Creatine kinase-MB (IU/L)	1.01 (1–1.01)	0		
Glucose (mmol/l)	1 (1–1)	0.1431		
BUN (mg/dL)	1.01 (1.01–1.02)	0		
Serum creatinine (mg/dL)	1.05 (0.95–1.16)	0.3065		
CAR	1.01 (1.01–1.01)	0	1.01 (1–1.01)	0.0071
Hemoglobin (g/l)	0.92 (0.86–0.99)	0.0211		
Hematocrit (%)	0.98 (0.96–1)	0.1051		
HDL (mmol/L)	1.01 (1–1.02)	0.0267	1.01 (1–1.02)	0.0026
Ferritin (mmol/L)	1 (1–1)	0		
Lactate (mmol/L)	1.11 (1.06–1.16)	0	1.1 (1.04–1.16)	0.001
Total bilirubin (mg/dL)	1.01 (0.98–1.05)	0.558		
Total protein (g/L)	0.81 (0.7–0.93)	0.0038		
Sodium (g/L)	0.99 (0.96–1.02)	0.5614		
Platelet (K/μL)	1 (1–1)	0.0026		
Potassium (g/L)	1.12 (0.91–1.38)	0.3027		
Chloride (g/L)	0.97 (0.95–1)	0.0606		
Total cholesterol (mEq/L)	1 (1–1)	0.3799		
NT-proBNP (pg/mL)	1 (1–1)	0	1 (1–1)	0.0001
SAPSII	1.04 (1.03–1.05)	0		
SOFA	1.16 (1.12–1.2)	0	1.16 (1.12–1.21)	0
Heart failure	0.56 (0.08–4.01)	0.5637		
Acute respiratory failure	1.84 (1.28–2.65)	0.0009		
Acute kidney failure	0.85 (0.6–1.19)	0.3402		
Acute liver failure	1.2 (0.49–2.93)	0.6921		
Septic shock	1.79 (1.27–2.52)	0.0008		
Stress ulcer	0 (0-Inf)	0.9923		
Toxic encephalopathy	0.73 (0.45–1.17)	0.1904		
Heparin	0.13 (0.07–0.23)	0	0.21 (0.11–0.39)	0
Amoxicillin clavulanic acid	0.07 (0.01–0.47)	0.0067	0.13 (0.02–0.94)	0.0432
Ampicillin sulbactam	0.27 (0.12–0.61)	0.0017		
Aripiprazole	0.53 (0.07–3.77)	0.5231		
Azithromycin	0.62 (0.39–1)	0.0504		
Aztreonam	0.82 (0.33–2)	0.658		
Cefazolin	0.1 (0.04–0.27)	0	0.16 (0.06–0.43)	0.0003
Cefepime	0.77 (0.54–1.09)	0.1358		
Cefpodoxime Proxetil	0.13 (0.02–0.9)	0.0389		
Ceftaroline	1.63 (0.52–5.13)	0.4011		
Ceftazidime	0.49 (0.29–0.85)	0.0104	0.38 (0.21–0.68)	0.0011
Ceftriaxone	0.51 (0.33–0.78)	0.0022	0.46 (0.29–0.73)	0.0009
Cephalexin	0.15 (0.04–0.6)	0.0076		
Ciprofloxacin HCl	0.33 (0.22–0.49)	0	0.45 (0.29–0.71)	0.0006
Clindamycin	0.69 (0.39–1.22)	0.2059		
Clotrimazole	0 (0-Inf)	0.9907		
Albumin 25%	0.82 (0.58–1.17)	0.2756		
Daptomycin	0.27 (0.12–0.61)	0.0016	0.39 (0.17–0.91)	0.0298
Dexamethasone	0.31 (0.15–0.6)	0.0006		
Dicloxacillin	0 (0-Inf)	0.9929		
Dobutamine	2.36 (1.36–4.1)	0.0024	2.12 (1.16–3.85)	0.0139
Dopamine	2.57 (1.58–4.18)	0.0001	2.24 (1.29–3.89)	0.004
Doxorubicin	0 (0-Inf)	0.9922		
Erythromycin	0.3 (0.12–0.73)	0.008	0.35 (0.14–0.9)	0.0291
Gentamicin	0.53 (0.26–1.09)	0.0828		
Hydrocortisone	0.88 (0.59–1.31)	0.5393		
Imipenem Cilastatin	0 (0-Inf)	0.993		
Insulin	0.65 (0.44–0.96)	0.0296		
Ketoconazole	0 (0-Inf)	0.9927		
Levofloxacin	0.58 (0.38–0.88)	0.0108		
Linezolid	0.39 (0.2–0.74)	0.0043		
Meropenem	0.76 (0.52–1.11)	0.155		
Metronidazole Flagyl	0.47 (0.31–0.72)	0.0004	0.49 (0.29–0.83)	0.008
Micafungin	1 (0.62–1.61)	0.9984		
Miconazole	0.32 (0.2–0.53)	0	0.4 (0.24–0.67)	0.0006
Nafcillin	0.84 (0.46–1.56)	0.5909		
Neomycin	2.09 (1.42–3.07)	0.0002		
Norepinephrine	1.15 (0.71–1.88)	0.5619		
Omeprazole	0.36 (0.24–0.56)	0	0.55 (0.35–0.86)	0.008
Pantoprazole	0.46 (0.33–0.65)	0	0.58 (0.4–0.85)	0.0054
Penicillin G potassium	0 (0-Inf)	0.9929		
Piperacillin Tazobactam	0.71 (0.5–1)	0.0518		
Racepinephrine	0.33 (0.05–2.35)	0.2673		
Triglyceride	1 (1–1)	0.2544		
Vancomycin	0.87 (0.46–1.67)	0.6811		
Voriconazole	0.15 (0.02–1.09)	0.0603		
Dextrose	1,214,154.79 (0–inf)	0.9926		

*ALP* alkaline phosphatase, *ALT* alanine aminotransferase, *AST* aspartate transaminase, *BUN* blood urea nitrogen, *HDL* high-density lipoprotein, *NT-proBNP* N-Terminal Pro-Brain Natriuretic Peptide, *RBC* red blood cell, *WBC* white blood cell, *SOFA* Sequential Organ Failure Assessment, *SAPS II* Simplified Acute Physiology Score II, *CRP* C-reactive protein, *ALB* serum albumin, *CAR* C-reactive protein/albumin ratio.

### Nomogram construction and validation

**Figure 3:**
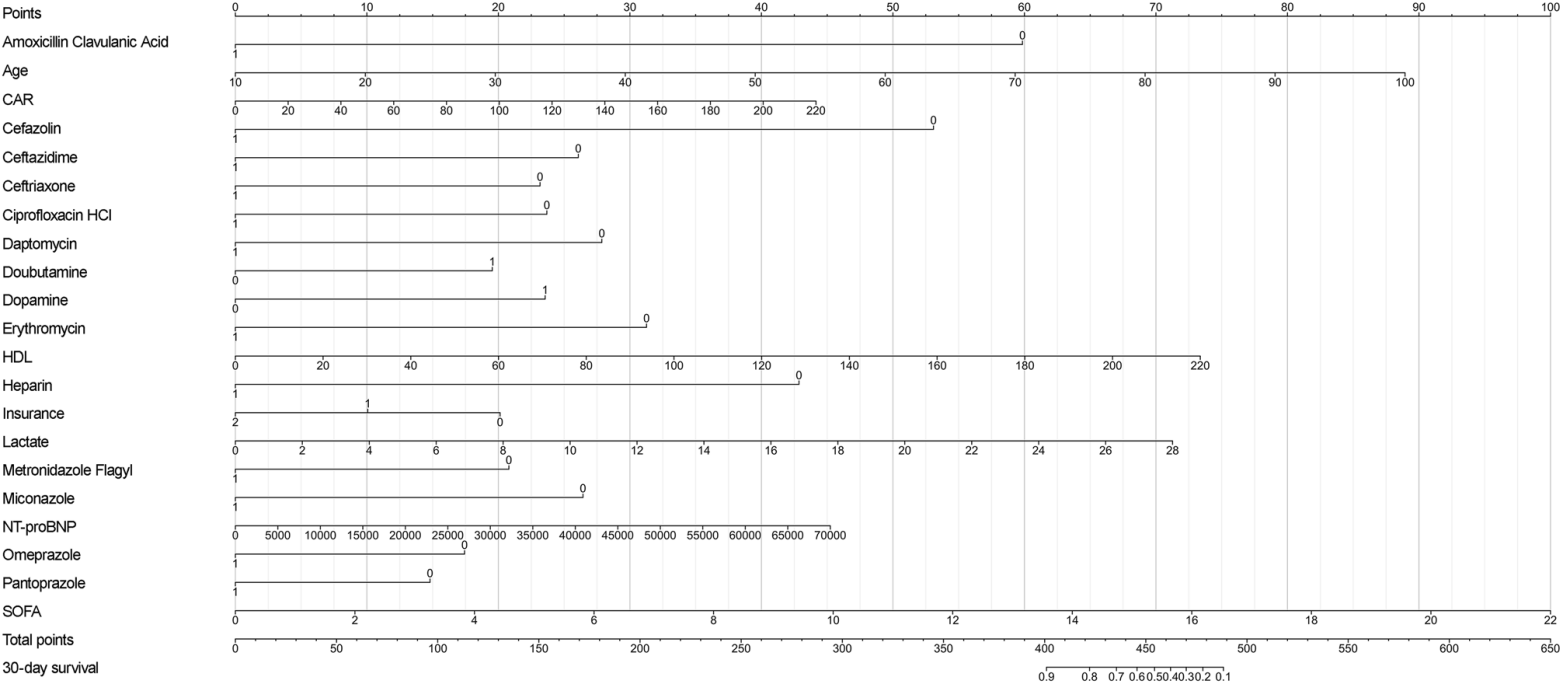
The nomogram for predicting 30-day survival for sepsis patients. The left column shows the points bar (top) and 21 parameters, with each to be scored with a vertical line to the points bar based on the different parameter values. The sum of the points is calculated (total points range, 0–650), and a vertical line is drawn from the total points bar to the 30-day survival probability below, to obtain survival probability of the patient. *CAR* C-reactive protein-to-albumin ratio, *HDL* high-density lipoprotein, *NT-proBNP* N-Terminal Pro-Brain Natriuretic Peptide, *SOFA* Sequential Organ Failure Assessment.

**Figure 4:**
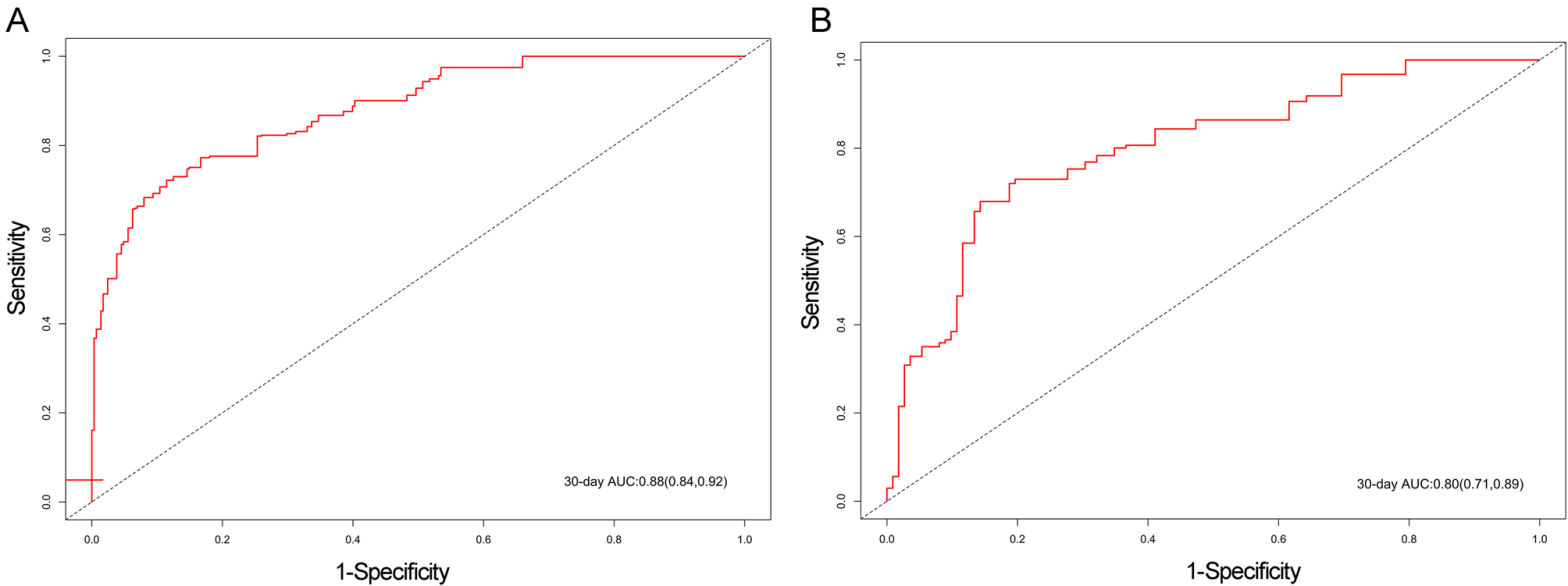
The 30-day Receiver operating characteristic (ROC) analysis in the training (**A**) and validation sets (**B**) for the nomogram. *AUC* area under the curve.

**Figure 5:**
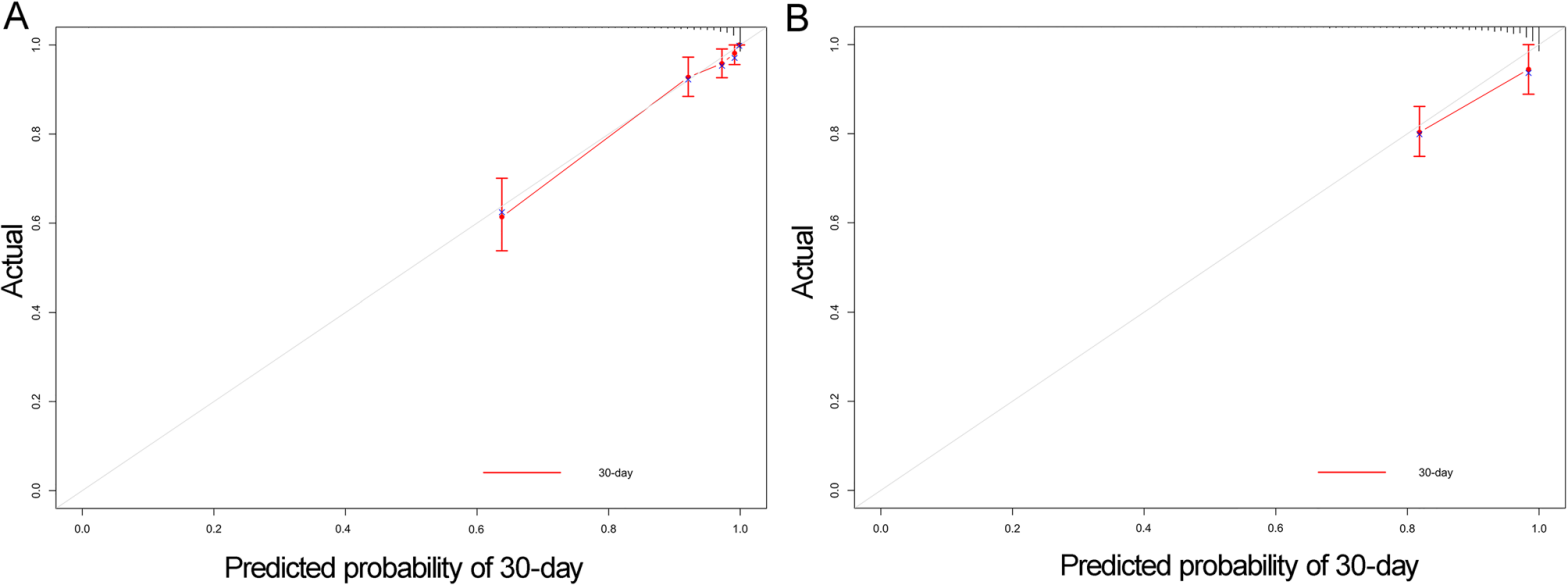
Calibration curve analysis for 30-day survival in the training (**A**) and validation cohorts (**B**). The abscissa (x-axis) represents the predicted survival rate and the ordinate (y-axis) represents the actual survival rate. The red dotted line is the reference line (predicted value equals the actual value), the solid grey line is the curve fitting line, and the error bars represent 95% confidence intervals. The calibration curves depict the agreement between predicted probabilities and observed outcomes.

Based on the results of multivariate Cox regression analysis, 23 independent prognostic factors, including age, race, insurance, marital status, NT-proBNP, HDL, lactate, CAR, SOFA, Amoxicillin Clavulanic Acid, Cefazolin, Ceftazidime, Ceftriaxone, Ciprofloxacin HCl, Daptomycin, Dobutamine, Dopamine, Erythromycin, Heparin, Metronidazole Flagyl, Miconazole, Omeprazole, Pantoprazole, were included in the construction of the nomogram for predicting the in-hospital death of sepsis patients, and the predictive model was also validated using the validation cohort (Fig. 3). The C-index of the model was 0.917 (SE: 0.112) for the training set and 0.935 (SE: 0.010) for the validation set, indicating that the nomogram has a good discriminative ability. Furthermore, 30-days ROC analysis showed that the AUC was 0.881 (95% CI: 0.840–0.924) in the training set and 0.798 (95% CI: 0.709–0.887) in the validation set (Fig. 4). Calibration curve analysis indicated that the nomogram has high prognostic accuracy and clinical value in both the training and validation cohorts (Fig. 5).

### Nomogram-based risk classification system

**Figure 6:**
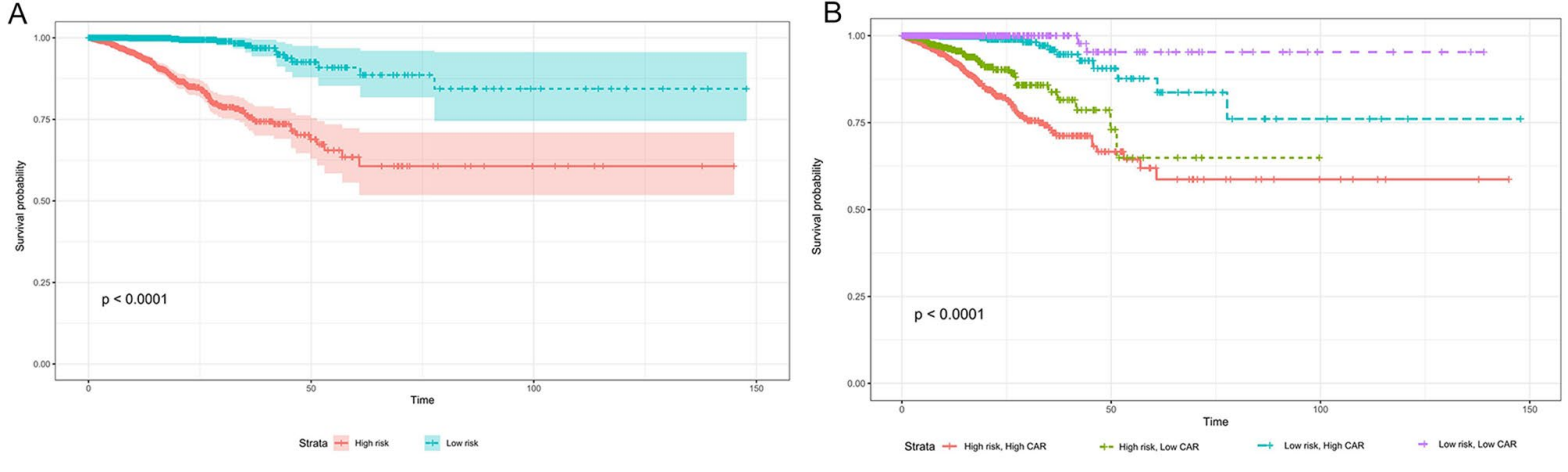
Kaplan–Meier curves for OS stratified by three risk groups in the sepsis patients (**A**) and Subgroup analysis of the correlation between CAR and OS in sepsis patients (**B**). Kaplan–Meier curves shows cumulative probability of OS according to groups at 30 days. *OS* overall survival, *CAR* C-reactive protein-to-albumin ratio.

The risk scores of sepsis patients were predicted by the nomogram, and the cutoff value was determined by median. Patients were divided into the low risk (total score ≤ 361.97) and high risk (total score > 361.97) groups. K–M analysis showed that the prognosis was significantly different between the two groups (*P* < 0.0001) (Fig. 6A). These data demonstrate that the risk stratification system based on the nomogram can accurately distinguish the survival of sepsis patients.

### Subgroup analysis

Subgroup analysis was performed to determine whether the correlation between CAR and OS in sepsis patients was stable across subgroups. When the stratified analysis was performed for CAR, the K–M curve showed that higher CAR values were associated with lower OS in each subgroup population (*P* < 0.0001) (Fig. 6B).

## Discussion

In this study, we showed that CAR was an independent prognostic factor for sepsis patients in the MIMIC-IV database and established a CAR-based risk-prediction nomogram. Our results demonstrated that age, race, insurance, marital status, NT-proBNP, HDL, lactate, CAR, SOFA, Amoxicillin Clavulanic Acid, Cefazolin, Ceftazidime, Ceftriaxone, Ciprofloxacin HCl, Daptomycin, Dobutamine, Dopamine, Erythromycin, Heparin, Metronidazole Flagyl, Miconazole, Omeprazole, and Pantoprazole were independent prognostic factors for sepsis patients. Analyses of C-index, ROC curve, and calibration curve showed that the CAR-based nomogram has good predictive performance in the prognosis of sepsis. In addition, the risk stratification system based on the nomogram further indicated that sepsis patients with high CAR had significantly higher in-hospital mortality than those with low CAR, which was further supported by the findings of the subgroup analysis. Taken together, these data indicate that our prediction nomogram has high clinical application value and can help clinicians accurately predict the prognosis of patients and make individualized treatment plans.

Sepsis has always been a major challenge in the clinical treatment of patients with severe infection^24^. Due to limited treatment methods, early identification and early intervention are the key to the treatment of sepsis^25^. Previous studies have investigated the predictive performance of several biomarkers in the prognosis of sepsis patients, including platelet^26^, neutrophil/lymphocyte ratio (NLR)^27^, lactate/albumin ratio^28^, plasminogen activator inhibitor-1 (PAI-1)^29^, signal peptide-CUB-epidermal growth factor-like domain-containing protein 1 (SCUBE-1)^30^, and vitamin D receptor^31^. CAR has been recently used as a new prognostic marker in COVID-19^32^, severe fever with thrombocytopenia syndrome (SFTS)^33^, and cardiac arrest^34^. However, the predictive performance of CAR in the prognosis of sepsis patients has not been reported.

Currently, the prevailing mechanism for sepsis pathophysiology is the balance theory of host response ^35^. In sepsis, the loss of balance in immune responses may lead to excessive inflammatory response characterized by the release of inflammatory factors, which in turn triggers systemic inflammation and multiple organ failure. Alternatively, immune imbalance may also result in anti-inflammatory hyperactivity and the production of anti-inflammatory cytokines, which can affect immune cell functions and cause the body to be in a state of imbalance. Thus, inflammatory factors are the most prominent markers throughout the development of sepsis. CRP is an acute phase reaction protein and a commonly used inflammatory marker in clinical practice ^36^. It was found that the CRP concentration is almost positively correlated with the degree of inflammation and tissue injury^37^. Many studies have reported that CRP is helpful in the diagnosis of inflammatory diseases, such as neonatal sepsis^38^, COVID-19^39^, pneumonia^40^, and influenza^41^. However, CRP alone cannot distinguish sepsis from SIRS due to its elevated level in both conditions^42^.

ALB is a protein synthesized by the liver and a conventional indicator for assessing the nutritional status of the body. In recent years, ALB has been shown to play an emerging role in human innate immunity by acting as a host defense agent during infection^43^. It was reported that ALB can serve as an independent predictor for in-hospital mortality in hospitalized patients over 90 years of age with acute infectious diseases^44^. Similarly, a retrospective cohort study reported that ALB was a predictor for the severity of abdominal sepsis in adult patients ^45^. Hypoalbuminemia is also commonly present in neonatal sepsis^46^, which is more likely to be associated with enhanced clearance from circulation rather than impaired synthesis by the liver ^47^.

A growing number of studies have pointed out that CAR plays a certain role in the evaluation and prognosis of heart disease^48^, cancer^49^, and infectious diseases. Elevated CAR level has been shown to be a reliable marker for infective endocarditis (IE) ^50^ and is associated with increased morbidity of spinal epidural abscess (SEA) ^51^. Moreover, the CAR level is also higher in complicated appendicitis than in simple appendicitis^52^. A recent study has concluded that CAR is an independent risk factor for pneumonia in middle-aged and older Finnish men^22^. In the case of neonatal sepsis, it is reported that CAR was an independent predictor for the presence and severity of neonatal sepsis, as well as the presence of sepsis in neonates with pneumonia^53,54^. In addition, CAR is an independent predictor for mortality in adult patients with severe sepsis and septic shock receiving early goal-directed therapy in the emergency department. Collectively, these findings indicate that CAR is a readily available and objective hematological biomarker for systemic inflammation.

To our knowledge, this is the first study to examine the relationship between CAR and sepsis in a relatively large population. Nevertheless, the study has several limitations. First, this study is a retrospective study with inherent biases that cannot characterize the association between CAR and morality in sepsis patients as well as prospective studies do. Therefore, further large-cohort multicenter prospective studies are warranted. Second, many clinical data that may be related to prognosis were unavailable due to the limitations of public databases. Third, we examined the relationship between mortality and the first measured CAR after admission, which did not allow us to assess the impact of dynamic CAR on prognosis. Last, our model was based on the MIMIC-IV database, which primarily contained U.S patients. Therefore, the generalizability of the model to the global population remains unclear.

## Conclusion

The CAR-based model we developed and validated has good predictive performance for the risk of in-hospital death in sepsis patients. This model can be used as a simple and practical tool for clinicians to timely and accurately identify sepsis patients at high risk of in-hospital death. Moreover, the model can provide a reference for disease risk stratification and support the development of prognostic treatment strategies and follow-up strategies for prolonging the survival of sepsis patients.

### Supplementary Information

Supplementary Table 1.

## Data Availability

All data generated and analysed during this study are included in this published article, and its supplementary information files.

## Abbreviations

CAR: C-reactive protein-to-albumin ratio
LASSO: Least absolute shrinkage and selection operator
ROC: Operating characteristic curve
OS: Overall survival
K-M: Kaplan–Meier
AUC: Area under the curve
CRP: C-reactive protein
ALB: Serum albumin
SOFA: Sequential Organ Failure Assessment
NLR: Neutrophil/lymphocyte ratio
PAI-1: Plasminogen activator inhibitor-1
SCUBE-1: Signal peptide-CUB-epidermal growth factor-like domain-containing protein 1
SFTS: Severe fever with thrombocytopenia syndrome
