# Supplementary material for: C-reactive protein-to-albumin ratio as a biomarker in patients with sepsis: a novel LASSO-COX based prognostic nomogram
Source: Sci Rep. 2023 Sep 15;13:15309. doi: 10.1038/s41598-023-42601-4 (PMC10504378; doi:10.1038/s41598-023-42601-4)
Supplement: Supplementary file 1 — Supplementary Table 1. [file 41598_2023_42601_MOESM1_ESM.docx]

**Supplement Table 1.** Supplement basic characters

| Factors | Define | Train(N=4489) | Test(N=1925) | All(N=6414) | t/Z(p) |
| --- | --- | --- | --- | --- | --- |
| Dextrose | No | 45(1) | 16(0.83) | 61(0.95) | 0.420(0.517) |
|  | Yes | 4444(99) | 1909(99.17) | 6353(99.05) |  |
| Insulin | No | 994(22.14) | 444(23.06) | 1438(22.42) | 0.658(0.417) |
|  | Yes | 3495(77.86) | 1481(76.94) | 4976(77.58) |  |
| Imipenem Cilastatin | No | 4413(98.31) | 1896(98.49) | 6309(98.36) | 0.291(0.590) |
|  | Yes | 76(1.69) | 29(1.51) | 105(1.64) |  |
| Neomycin | No | 3908(87.06) | 1683(87.43) | 5591(87.17) | 0.166(0.684) |
|  | Yes | 581(12.94) | 242(12.57) | 823(12.83) |  |
| Vancomycin | No | 513(11.43) | 211(10.96) | 724(11.29) | 0.293(0.588) |
|  | Yes | 3976(88.57) | 1714(89.04) | 5690(88.71) |  |
| Cefpodoxime Proxetil | No | 4065(90.55) | 1733(90.03) | 5798(90.4) | .434(0.51) |
|  | Yes | 424(9.45) | 192(9.97) | 616(9.6) |  |
| Ceftaroline | No | 4435(98.8) | 1901(98.75) | 6336(98.78) | .022(0.883) |
|  | Yes | 54(1.2) | 24(1.25) | 78(1.22) |  |
| Cefepime | No | 1994(44.42) | 871(45.25) | 2865(44.67) | .373(0.541) |
|  | Yes | 2495(55.58) | 1054(54.75) | 3549(55.33) |  |
| Cephalexin | No | 3969(88.42) | 1711(88.88) | 5680(88.56) | .290(0.59) |
|  | Yes | 520(11.58) | 214(11.12) | 734(11.44) |  |
| Ketoconazole | No | 4366(97.26) | 1858(96.52) | 6224(97.04) | 2.570(0.109) |
|  | Yes | 123(2.74) | 67(3.48) | 190(2.96) |  |
| Dicloxacillin | No | 4446(99.04) | 1902(98.81) | 6348(98.97) | .742(0.389) |
|  | Yes | 43(0.96) | 23(1.19) | 66(1.03) |  |
| Norepinephrine | No | 4100(91.33) | 1809(93.97) | 5909(92.13) | 12.942(0.000) |
|  | Yes | 389(8.67) | 116(6.03) | 505(7.87) |  |
| Gentamicin | No | 4102(91.38) | 1768(91.84) | 5870(91.52) | .376(0.54) |
|  | Yes | 387(8.62) | 157(8.16) | 544(8.48) |  |
| Hydrocortisone | No | 3511(78.21) | 1504(78.13) | 5015(78.19) | .006(0.941) |
|  | Yes | 978(21.79) | 421(21.87) | 1399(21.81) |  |
| Piperacillin Tazobactam | No | 2549(56.78) | 1102(57.25) | 3651(56.92) | .118(0.731) |
|  | Yes | 1940(43.22) | 823(42.75) | 2763(43.08) |  |
| Penicillin G Potassium | No | 4416(98.37) | 1893(98.34) | 6309(98.36) | .011(0.917) |
|  | Yes | 73(1.63) | 32(1.66) | 105(1.64) |  |
| Levofloxacin | No | 3033(67.57) | 1304(67.74) | 4337(67.62) | .019(0.891) |
|  | Yes | 1456(32.43) | 621(32.26) | 2077(32.38) |  |
| Nafcillin | No | 4158(92.63) | 1797(93.35) | 5955(92.84) | 1.064(0.302) |
|  | Yes | 331(7.37) | 128(6.65) | 459(7.16) |  |
| Micafungin | No | 4089(91.09) | 1769(91.9) | 5858(91.33) | 1.108(0.293) |
|  | Yes | 400(8.91) | 156(8.1) | 556(8.67) |  |
| Meropenem | No | 3423(76.25) | 1502(78.03) | 4925(76.79) | 2.376(0.123) |
|  | Yes | 1066(23.75) | 423(21.97) | 1489(23.21) |  |
| Clotrimazole | No | 4165(92.78) | 1786(92.78) | 5951(92.78) | .000(0.996) |
|  | Yes | 324(7.22) | 139(7.22) | 463(7.22) |  |
| Clindamycin | No | 3834(85.41) | 1671(86.81) | 5505(85.83) | 2.160(0.142) |
|  | Yes | 655(14.59) | 254(13.19) | 909(14.17) |  |
| Voriconazole | No | 4385(97.68) | 1891(98.23) | 6276(97.85) | 1.940(0.164) |
|  | Yes | 104(2.32) | 34(1.77) | 138(2.15) |  |
| Albumin25% | No | 2905(64.71) | 1231(63.95) | 4136(64.48) | .345(0.557) |
|  | Yes | 1584(35.29) | 694(36.05) | 2278(35.52) |  |
| Aztreonam | No | 4338(96.64) | 1850(96.1) | 6188(96.48) | 1.123(0.289) |
|  | Yes | 151(3.36) | 75(3.9) | 226(3.52) |  |
| Ampicillin Sulbactam | No | 3758(83.72) | 1624(84.36) | 5382(83.91) | .419(0.517) |
|  | Yes | 731(16.28) | 301(15.64) | 1032(16.09) |  |
| Azithromycin | No | 3264(72.71) | 1359(70.6) | 4623(72.08) | 2.991(0.084) |
|  | Yes | 1225(27.29) | 566(29.4) | 1791(27.92) |  |
| Doxorubicin | No | 4430(98.69) | 1892(98.29) | 6322(98.57) | 1.524(0.217) |
|  | Yes | 59(1.31) | 33(1.71) | 92(1.43) |  |
| Aripiprazole | No | 4428(98.64) | 1903(98.86) | 6331(98.71) | .492(0.483) |
|  | Yes | 61(1.36) | 22(1.14) | 83(1.29) |  |
| Racepinephrine | No | 4418(98.42) | 1881(97.71) | 6299(98.21) | 3.793(0.051) |
|  | Yes | 71(1.58) | 44(2.29) | 115(1.79) |  |
